# Supplementary material for: Phylogeny-corrected identification of microbial gene families relevant to human gut colonization
Source: PLoS Comput Biol. 2018 Aug 9;14(8):e1006242. doi: 10.1371/journal.pcbi.1006242 (PMC6084841; doi:10.1371/journal.pcbi.1006242)
Supplement: S1 Appendix — Reference material giving definitions of mathematical symbols used in the Methods. (PDF) [file pcbi.1006242.s001.pdf]

Glossary of notation

- Data and metadata
  - A: $i \times j$ binary matrix of microbial presence-absence, where i is the number of microbes, j is the number of samples, and $a_{m,n}$ is 1 when microbe m is observed in sample n and 0 otherwise
  - $a_{m,N}$: a vector of presence-absences for microbe m in samples $n \in N$
  - e_x , d_y: environment x or study population y, each corresponding to a set of samples
  - $E = \{e_1, \ldots, e_k\}$: the set of environments being studied or compared (e.g., body sites; health vs. disease)
  - $D = \{d_1, ..., d_l\}$: the set of study populations
- Linear models
  - $\vec{\phi}_{x,E[,D]}(A)$: phenotype vector, calculated for environment $e_x \in E$ and potentially adjusting for dataset effects D, based on microbial presence-absence matrix A, with elements corresponding to phenotype estimates for individual microbes
  - $\vec{\phi}^{\text{Prev}}_{x,E,D}(A)$: prevalence phenotype estimates (based on $\text{logit}(\hat{p}^{\text{ADDW}}_{m,N,D}(A))$; see below).
  - $\vec{\phi}^{\text{Spec}}_{x,E}(A)$: environmental specificity score phenotype estimates (based on $\text{logit}(\widehat{s}^{\text{MAP}}_{m,x,E}(A; b))$; see below).
  - $\beta_{0,g}$: in the linear model for gene g, intercept term used to model the average value of a given phenotype $\vec{\phi}_{x,E}(A)$
  - $\beta_{1,g}$: in the linear model for gene g, the effect of having vs. not having gene g on a given phenotype $\vec{\phi}_{x,E}(A)$
  - $\vec{I}_g$: the binary vector of gene presence-absence whose elements are $I_{g,m}$, equal to 0 if the gene g is absent in microbe m and 1 if it is present
- Phenotype estimation
  - $p_{m,N}$: prevalence, the probability of observing a microbe m in a set of samples N $P(m|N)$
  - $\hat{p}^{\text{MLE}}_{m,N}(A)$: the maximum-likelihood estimate of prevalence, based on the presence-absence matrix A
  - $\hat{p}^{\text{ADD}}_{m,N}(A)$: an estimate of prevalence based on the presence-absence matrix A using additive smoothing
  - $\hat{p}^{\text{ADDW}}_{m,N,D}(A)$: an estimate of prevalence based on the presence-absence matrix A using additive smoothing, and additionally weighting by the inverse number of samples per dataset in D
  - $\hat{p}^{\text{EnvW}}_{m,E}(A)$: an estimate of the prevalence across environments, weighted by their probability (i.e., $P(m)$ obtained by marginalizing $P(m|e_x)$)
  - $s_{m,x,E}$: environment specificity, the probability of being in a particular environment e_x given that microbe m was observed $P(e_x|m)$
  - $\hat{s}_{m,x,E}(A)$: an estimate of environment specificity based on presence-absence matrix A
  - b: a hyperparameter controlling the width of the Laplace prior on $\hat{s}_{m,x,E}(A)$ (i.e., the amount of shrinkage in the estimate)
  - b_{optim}: a value of b optimized for sensitivity and specificity in parametric simulations
  - $P(e_x)$: the prior probability of encountering environment e_x; we use either an uninformative uniform prior (for bodysites), or take this prior from epidemiological data (for disease comparisons)
  - $\widehat{s}^{\text{MAP}}_{m,x,E}(A; b)$: a maximum a posteriori (MAP) estimate of environment specificity score for environment e_x based on presence-absence matrix A and the shrinkage hyperparameter b; in this paper we calculate environmental specificity scores for $x = \text{CD}$ (Crohn's disease specificity) and $x = \text{Gut}$ (healthy gut specificity)

- Simulation and censoring analysis
  - $\vec{\phi}^{\text{Sim}}$: a simulated continuous phenotype
  - $\vec{I}^{\text{Sim}}$: a simulated binary genotype (gene presence-absence)
  - α: Ives-Garland α, the sum of the transition probabilities from 0 to 1 and from 1 to 0 in a Markov model of binary trait evolution across a tree (i.e., a measure of phylogenetic signal in a binary trait)
  - β_0: assigned parameter giving the ancestral state of the simulated genotype $\vec{\phi}^{\text{Sim}}$
  - β_1: assigned parameter giving the degree to which the continuous phenotype $\vec{\phi}^{\text{Sim}}$affects the binary genotype $\vec{I}^{\text{Sim}}$; a measure of effect size of *phenotype on gene*
  - $\hat{\beta}_0^{\text{Prev}}$: the estimated ancestral state of our prevalence phenotype $\vec{\phi}_{x,E,D}^{\text{Prev}}(A)$, using a Brownian motion model of trait evolution
  - $\widehat{\sigma^2}$: the estimated (non-phylogenetic) variance of our prevalence phenotype $\vec{\phi}_{x,E,D}^{\text{Prev}}(A)$
  - $\hat{\beta}_1^{\text{Test}}$: estimated value of effect of *gene on phenotype* from the phylogenetic linear model
  - F: ratio of prevalences, comparing taxa with a given gene (numerator) to taxa without (denominator); an alternative measure of effect size of *gene on phenotype*
  - $\hat{\beta}_1^c$: when using a bootstrap null, estimated value of effect of *null gene on phenotype* from the phylogenetic linear model
  - $\vec{\phi}^{\text{Unc}^c}$: a version of the phenotype $\vec{\phi}$ where values at the certain limit of detection have been imputed based on a truncated normal
  - $\vec{T}^c$: a vector the same length as $\vec{\phi}$ whose elements have been randomly drawn from a truncated normal distribution
  - $\hat{\beta}_1^{\text{Test}^c}$: an estimated value of effect of *gene on the mock-uncensored phenotype* $\vec{\phi}^{\text{Unc}^c}$
  - K: the value at which left-censoring starts for a phenotype $\vec{\phi}$, expressed as standard deviations below the mean
- Compositionality and prevalence accuracy analysis
  - B_i: an $||M|| \times ||N||$ matrix with elements $b_{(m,n)_i}$, corresponding to the abundance of microbe m in sample n and simulation i
    - B_i^{Abs}: absolute abundances
    - B_i^{relCN}: relative abundances obtained by column-normalizing B_i^{Abs}
    - B_i^{Counts}: counts per microbe per sample, obtained by Dirichlet-Multinomial sampling
    - B_i^{relDM}: relative abundances obtained by column-normalizing B_i^{Counts}
  - $c_{m,n}$: a binary random variable determining whether a microbe m was truly present in a given sample n
  - μ_m: a microbe-specific average abundance
  - z_m: a microbe-specific prevalence
  - θ_{n_i}: multinomial probability parameters for a given sample n in simulation i
  - r_n: the number of total reads in a sample n
  - α_m: a Dirichlet parameter for a particular microbe m
