# Supplementary figures and images for: Phylogeny-corrected identification of microbial gene families relevant to human gut colonization
Source: PLoS Comput Biol. 2018 Aug 9;14(8):e1006242. doi: 10.1371/journal.pcbi.1006242 (PMC6084841; doi:10.1371/journal.pcbi.1006242)

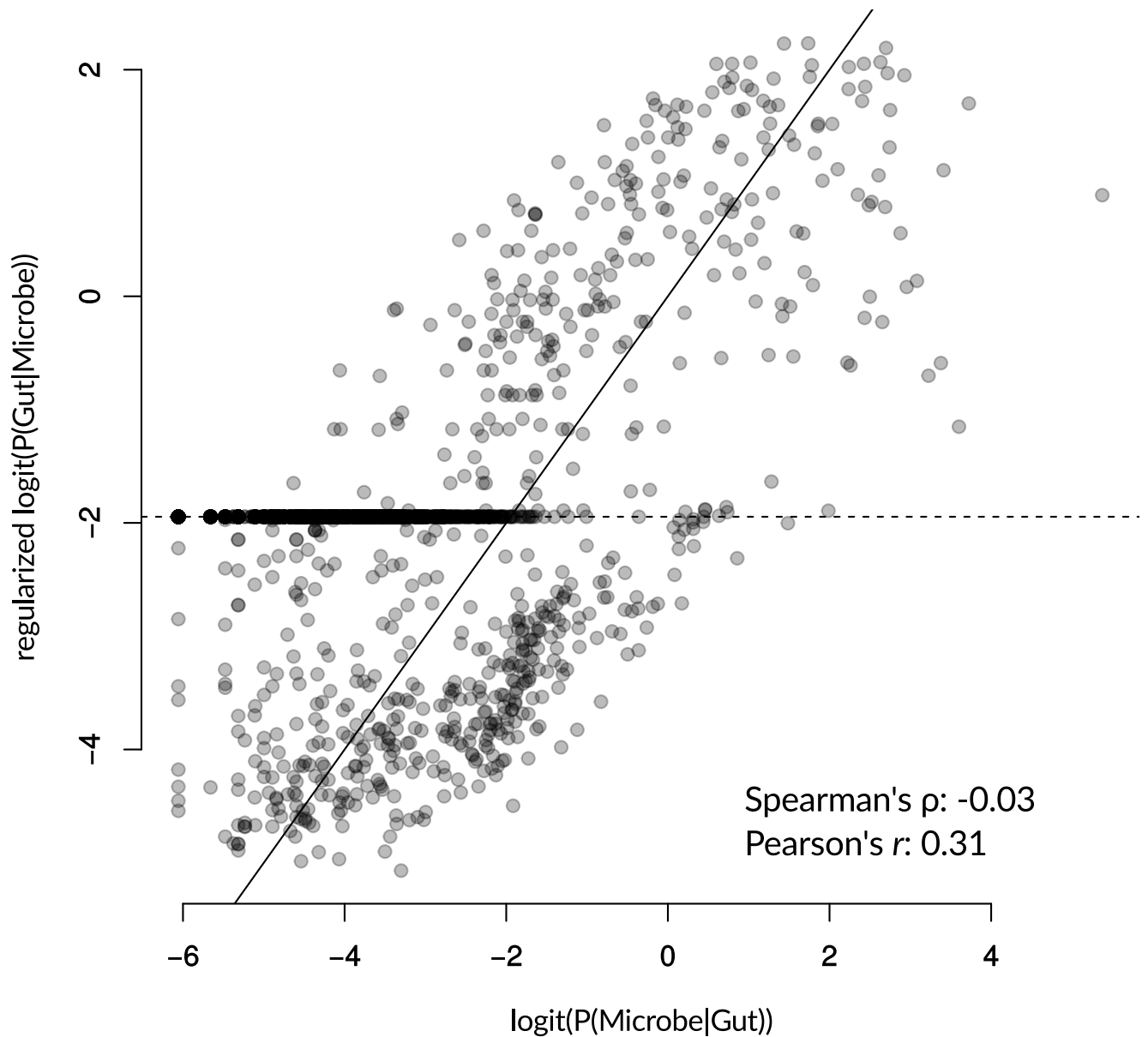

Supplement: S1 Fig — (PDF) [file pcbi.1006242.s006.pdf]

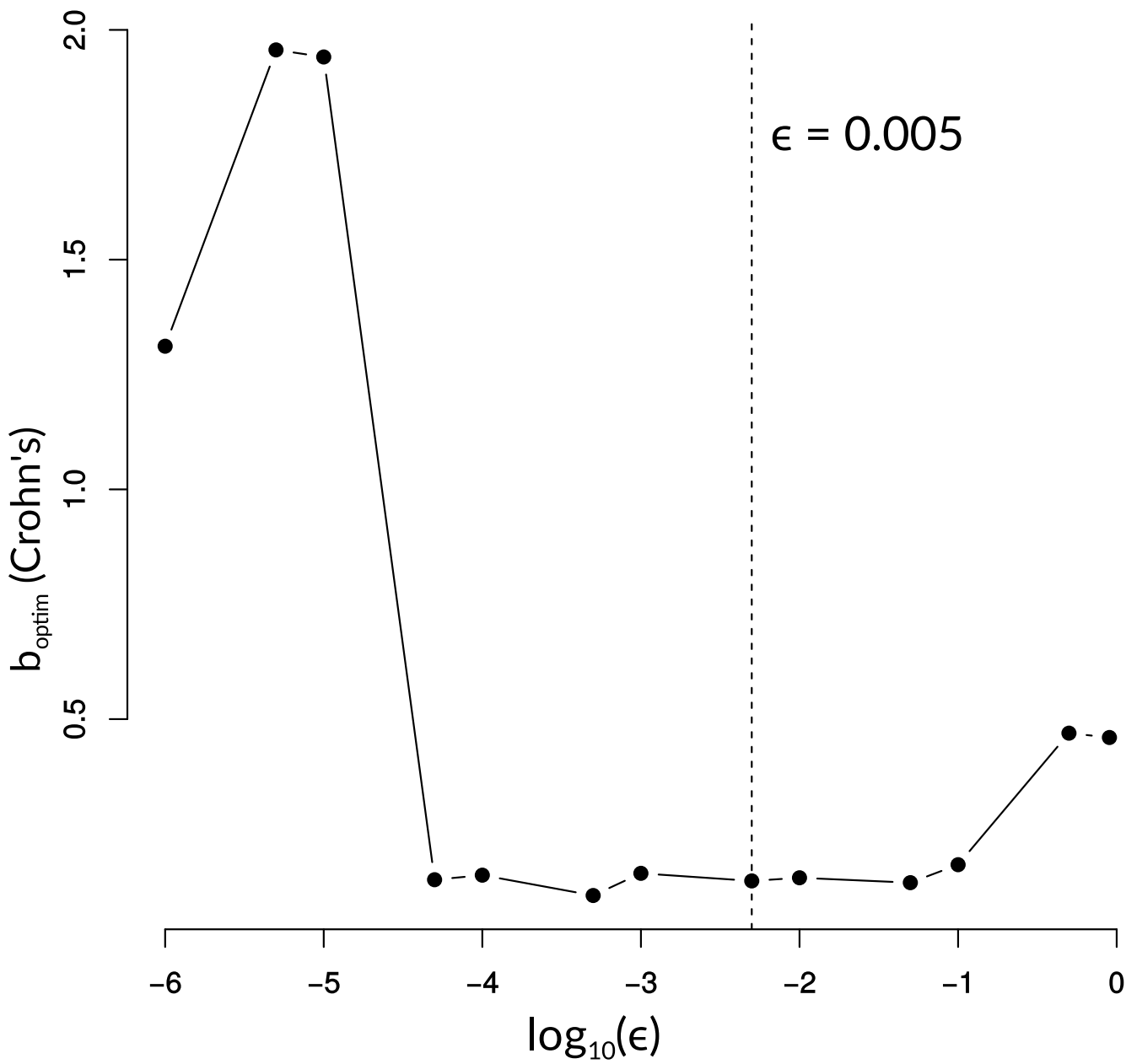

Supplement: S4 Fig — Y-axis gives the best boptim value obtained given a particular log10(ϵ) selected when performing Laplace shrinkage of s^m,CD,EMAP(A) estimates. The value of ϵ used in the manuscript (0.005) is highlighted with a vertical dashed line. (PDF) [file pcbi.1006242.s009.pdf]

# Glutamate/gamma-aminobutyrate antiporter (FIG01387141)

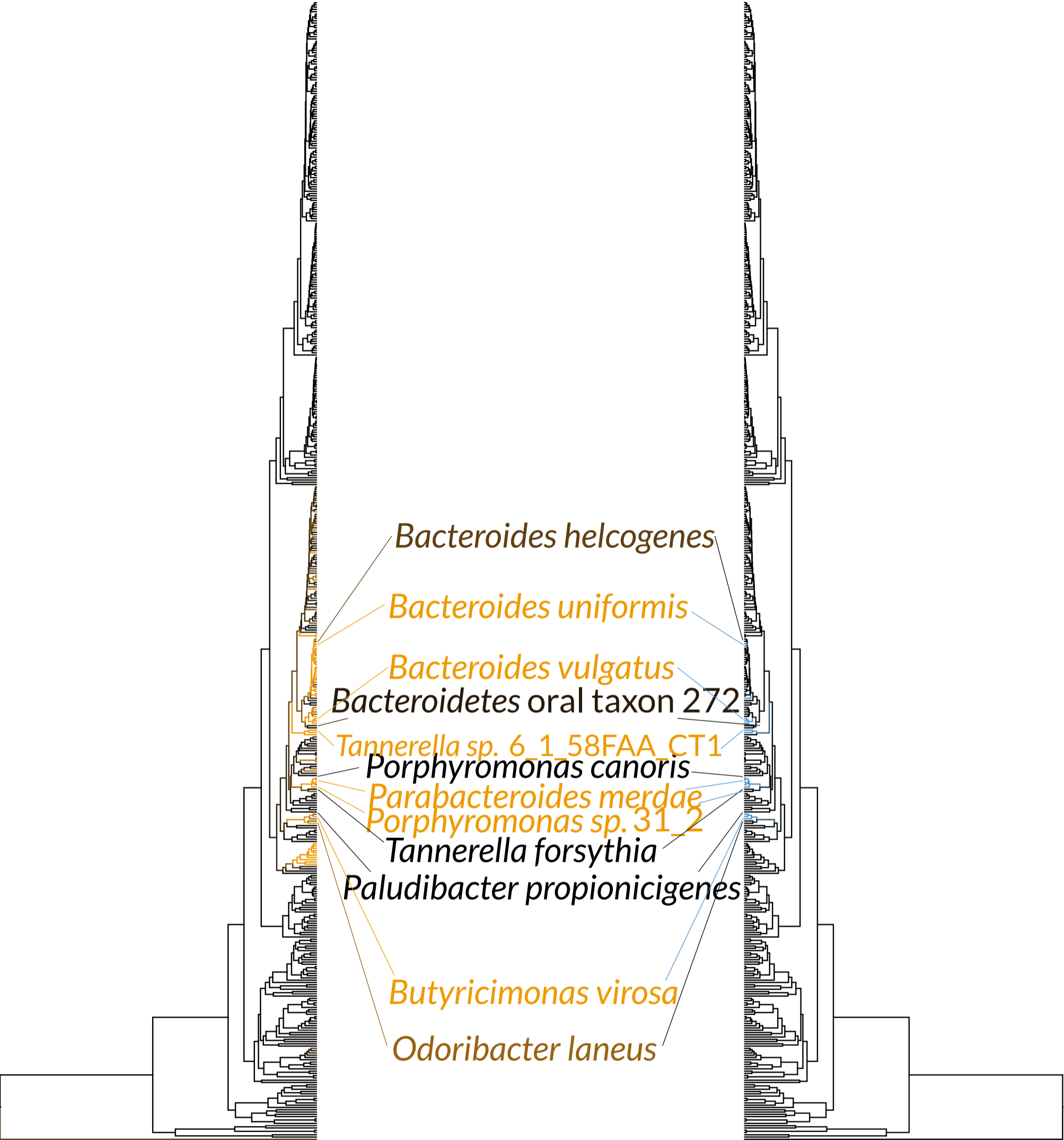

logit-prevalence

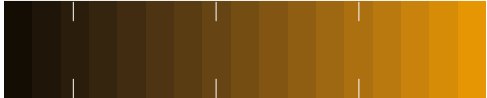

-4 -3 -2

gene

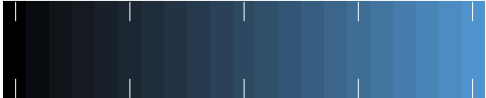

0.00 0.25 0.50 0.75 1.00

Supplement: S5 Fig — As in Fig 2, the tree on the left is colored by species prevalence (black to orange), while the tree on the right is colored by gene presence-absence (blue to black), with selected species called out in the middle, and lines linking species labels to leaves that match leaf color. (PDF) [file pcbi.1006242.s010.pdf]

estimated

best fit

uncensored

density

type

estimated

best fit

uncensored

 $K$ 

0.5

0.4

0.3

0.2

0.1

0.0

-10 -5 0 5

-10 -5 0 5

-10 -5 0 5

logit-prevalence

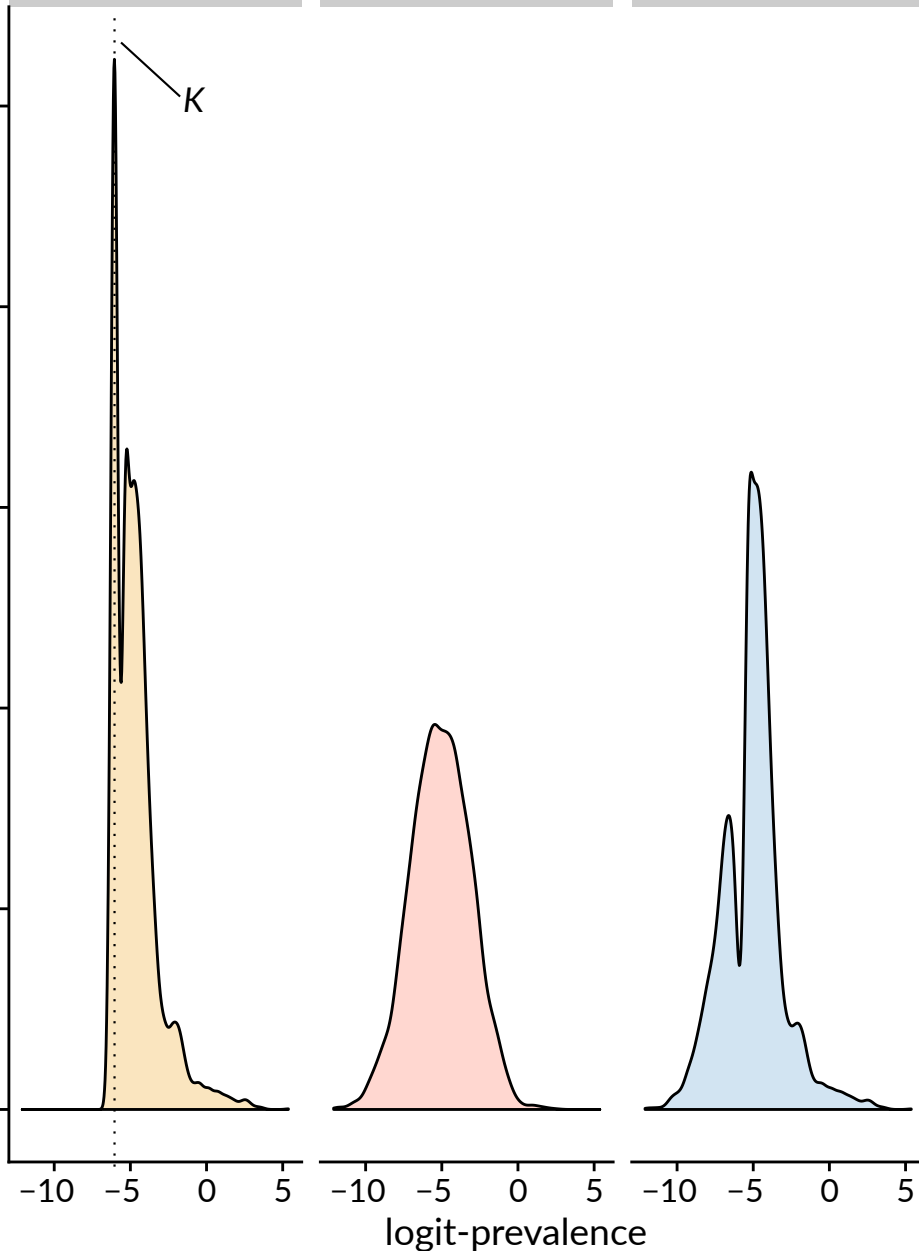

Supplement: S6 Fig — A) Density of estimated logit-prevalence distribution, ϕ→x,E,DPrev(A), showing pile-up of values at the limit of detection K. B) Density of a normal distribution with mean and standard deviation obtained from best-fit of truncated normal to ϕ→x,E,DPrev(A). C) “Uncensored” version of ϕ→x,E,DPrev(A). Data points at or below K have been replaced by random sampling from a truncated normal, with mean and standard deviation as in B and with K as upper truncation point. (PDF) [file pcbi.1006242.s011.pdf]

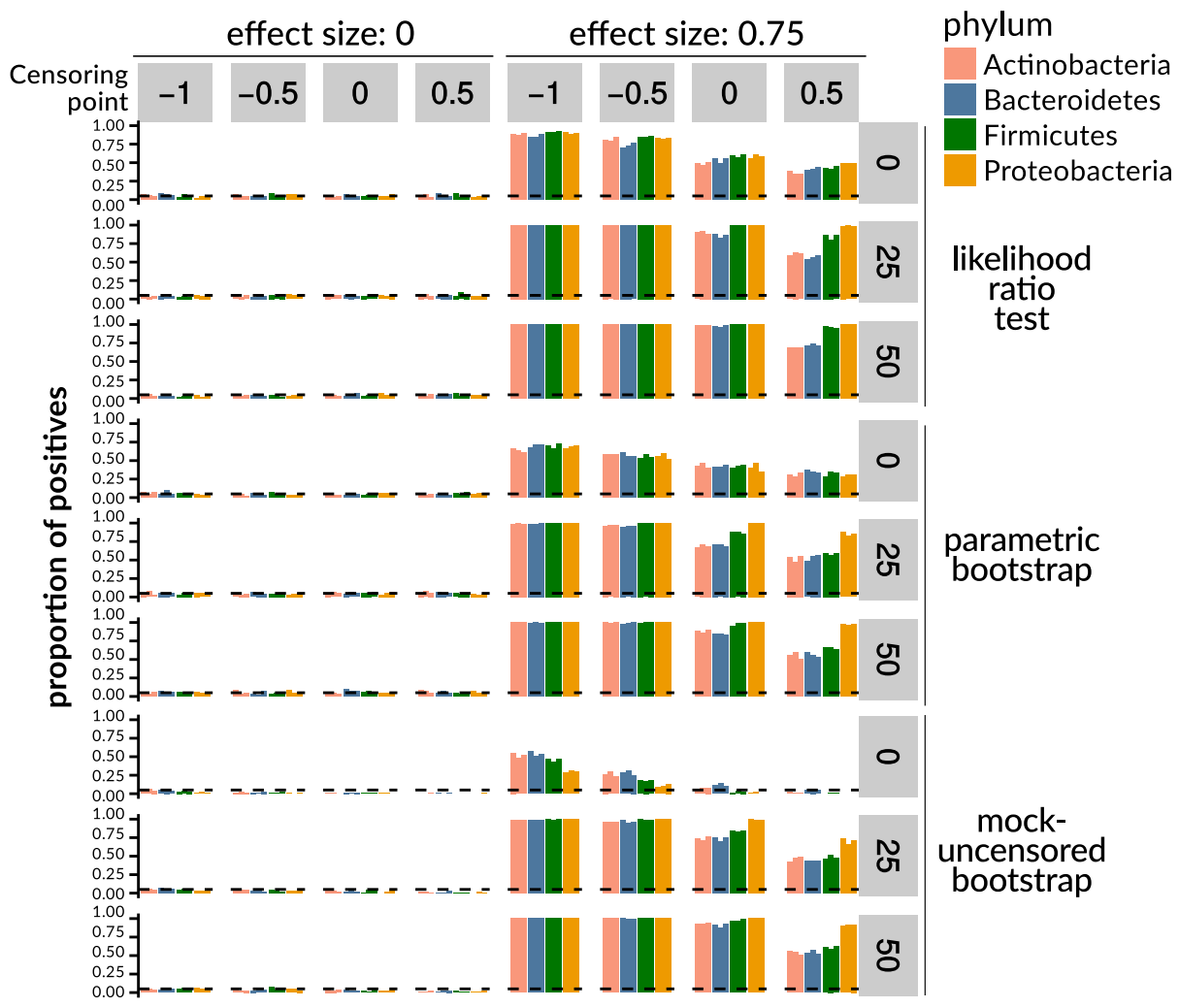

Supplement: S7 Fig — Bars give replicate measurements of false positive rate (left, effect size of 0) and power (right, effect size of 0.75) across the different phyla (colors), based on simulating binary genotypes and continuous phenotypes as in Methods, “Power analysis”, with varying levels of left-censoring (“censoring point”), and obtaining p-values with the three methods described in Methods, “Assessing the potential impact of sampling with left-censoring.” Horizontal dashed lines give a rate of 0.05. Binary genotypes had varying levels of Ives-Garland α (0, 25, 50), representing high to low phylogenetic signal. (PDF) [file pcbi.1006242.s012.pdf]

Bacteroidetes

Firmicutes

Proteobacteria

Actinobacteria

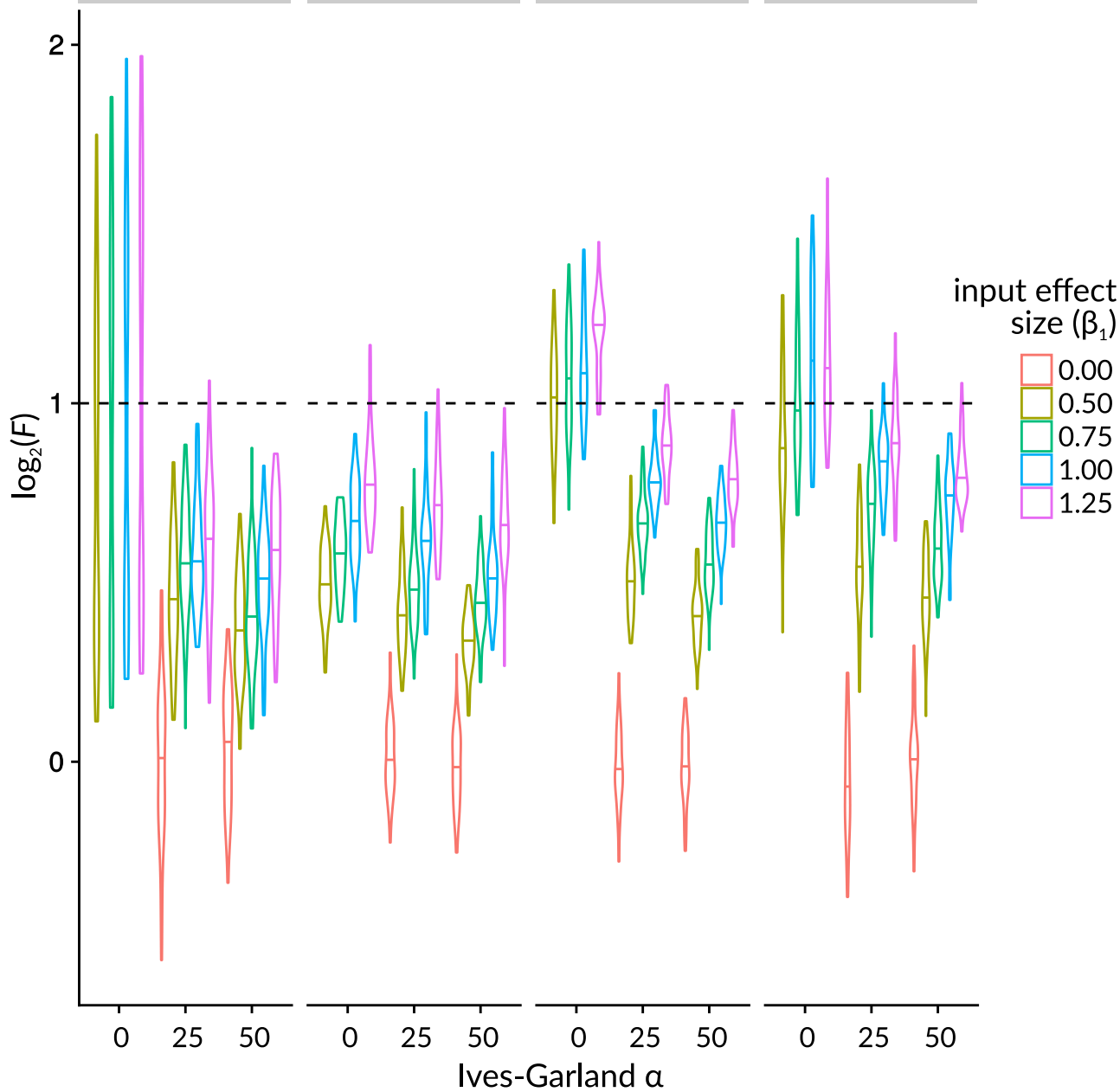

Supplement: S8 Fig — To give a more intuitive sense of scale for simulated effect sizes, simulations were performed as in Methods, “Power analysis”, with effect sizes β1 ranging from 0 to 1.25. After fitting phylogenetic models to the simulated phenotypes and genotypes, the average prevalences with the simulated gene, logistic(β1,g + β0,g), and without, logistic(β0,g), were computed, and their ratio F was taken. log2(F) is plotted here, such that a value of 1 means the gene conferred (on average) a 2-fold change in prevalence. Violin plots were made of 50 simulations. (PDF) [file pcbi.1006242.s013.pdf]

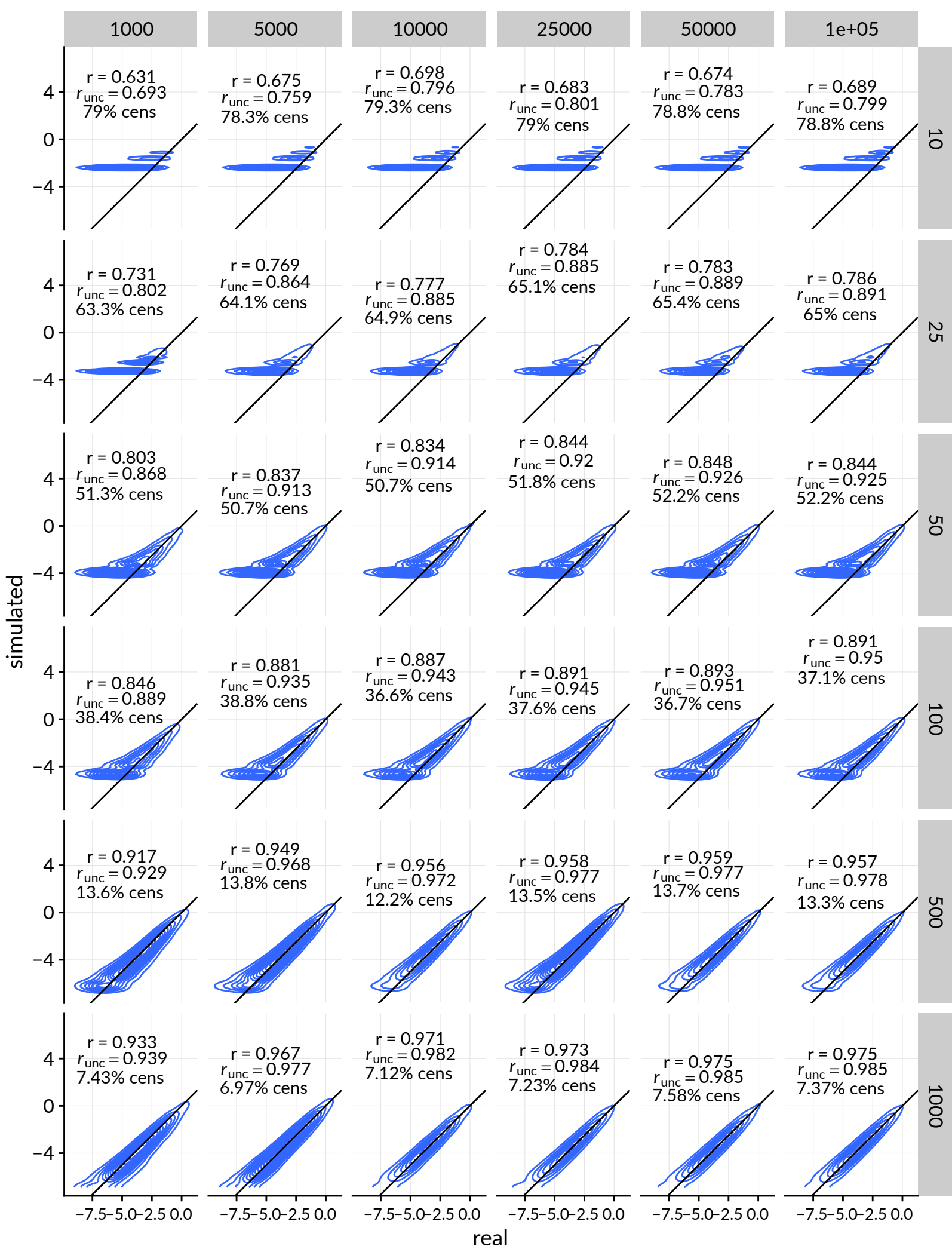

Supplement: S10 Fig — We simulated microbiome data using Dirichlet-Multinomial sampling combined with zero inflation. Here, we assumed that true prevalences were distributed logit(zm)∼N(-4,2). We then plotted (blue contours) the true logit-prevalence zm (x-axis) versus the calculated logit-prevalence from the simulation logit(p^m,N), for varying geometric-mean read depths rn (columns) and sample sizes ||N|| (rows). The values given for each sample size and read depth are the Pearson correlation r, the Pearson correlation over non-censored microbes only runc, and the percent of microbes with censored prevalences (“cens”). (PDF) [file pcbi.1006242.s015.pdf]

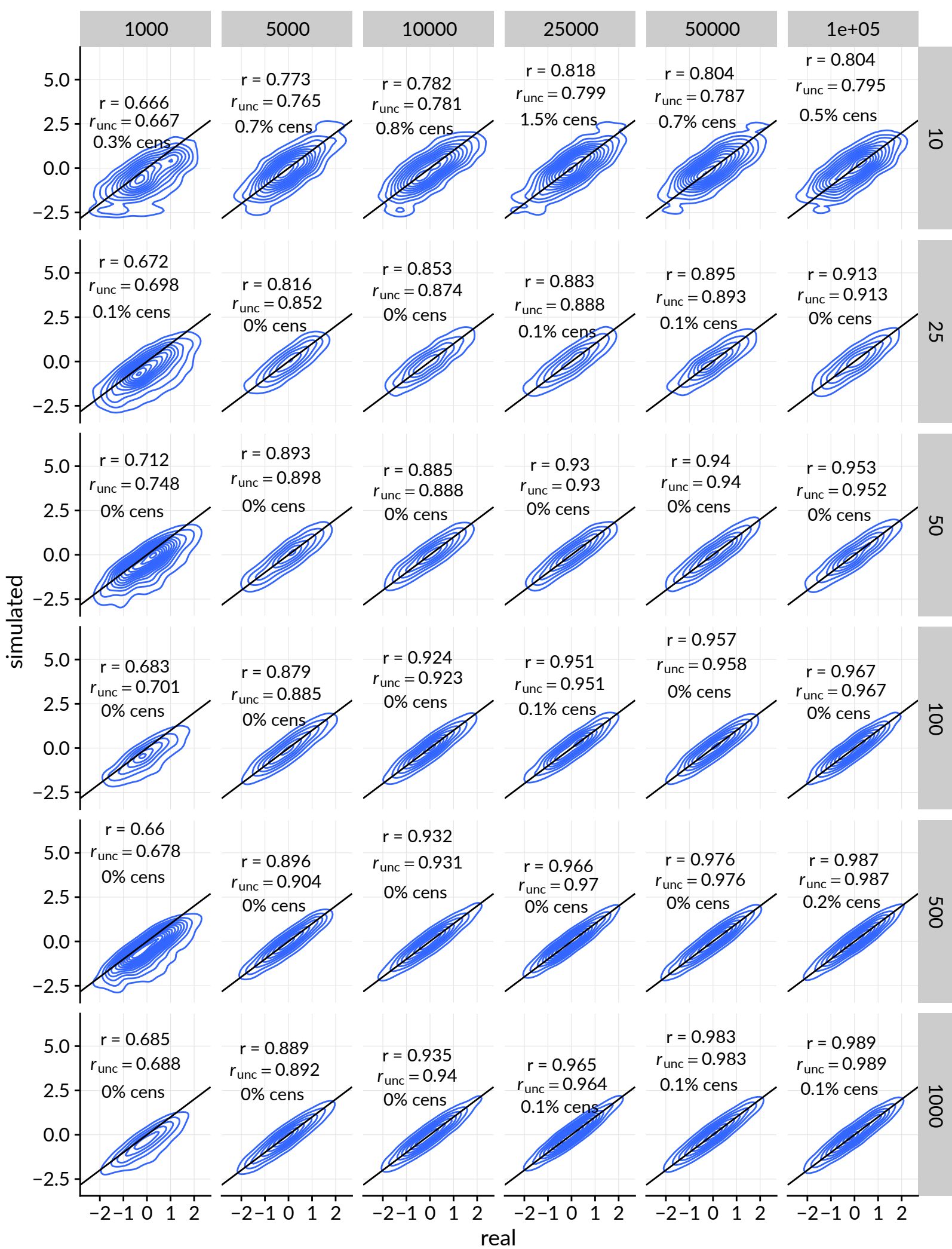

Supplement: S11 Fig — As in S10 Fig, but with logit(zm)∼N(0,1). (PDF) [file pcbi.1006242.s016.pdf]
