# Supplementary material for: Phylogeny-corrected identification of microbial gene families relevant to human gut colonization
Source: PLoS Comput Biol. 2018 Aug 9;14(8):e1006242. doi: 10.1371/journal.pcbi.1006242 (PMC6084841; doi:10.1371/journal.pcbi.1006242)
Supplement: S2 Fig — Using MIDAS, we calculate species relative abundances from shotgun sequencing data. These are binarized to yield a matrix of microbial presence/absence, with rows corresponding to microbes and columns corresponding to samples. Samples are organized into environments (i.e., the environments from which the sample was collected) and into datasets (corresponding to samples collected as part of the same project). Using the presence/absence matrix together with these metadata, we estimate phenotype vectors ϕ→(A), whose elements are estimates of microbial phenotypes. These phenotypes fall into two groups: prevalence (ϕ→x,E,DPrev(A)) and environmental specificity scores (ϕ→x,ESpec(A)). Separately, we use the whole genomes incorporated into the MIDAS database to assemble a matrix of gene presence/absence in the pangenome of microbes, and to construct a phylogenetic species tree based on previously-validated single-copy marker genes. We subset this tree to yield four phylum-specific trees. The inputs to our phylogenetic models are a phenotype vector, a gene presence-absence vector, and a phylogenetic tree. Based on these models, we estimate p-values for a non-zero effect of the gene on the phenotype, then convert these p-values into q-values to obtain predicted gene-phenotype interactions at a given false discovery rate (here, 5%). (PDF) [file pcbi.1006242.s007.pdf]

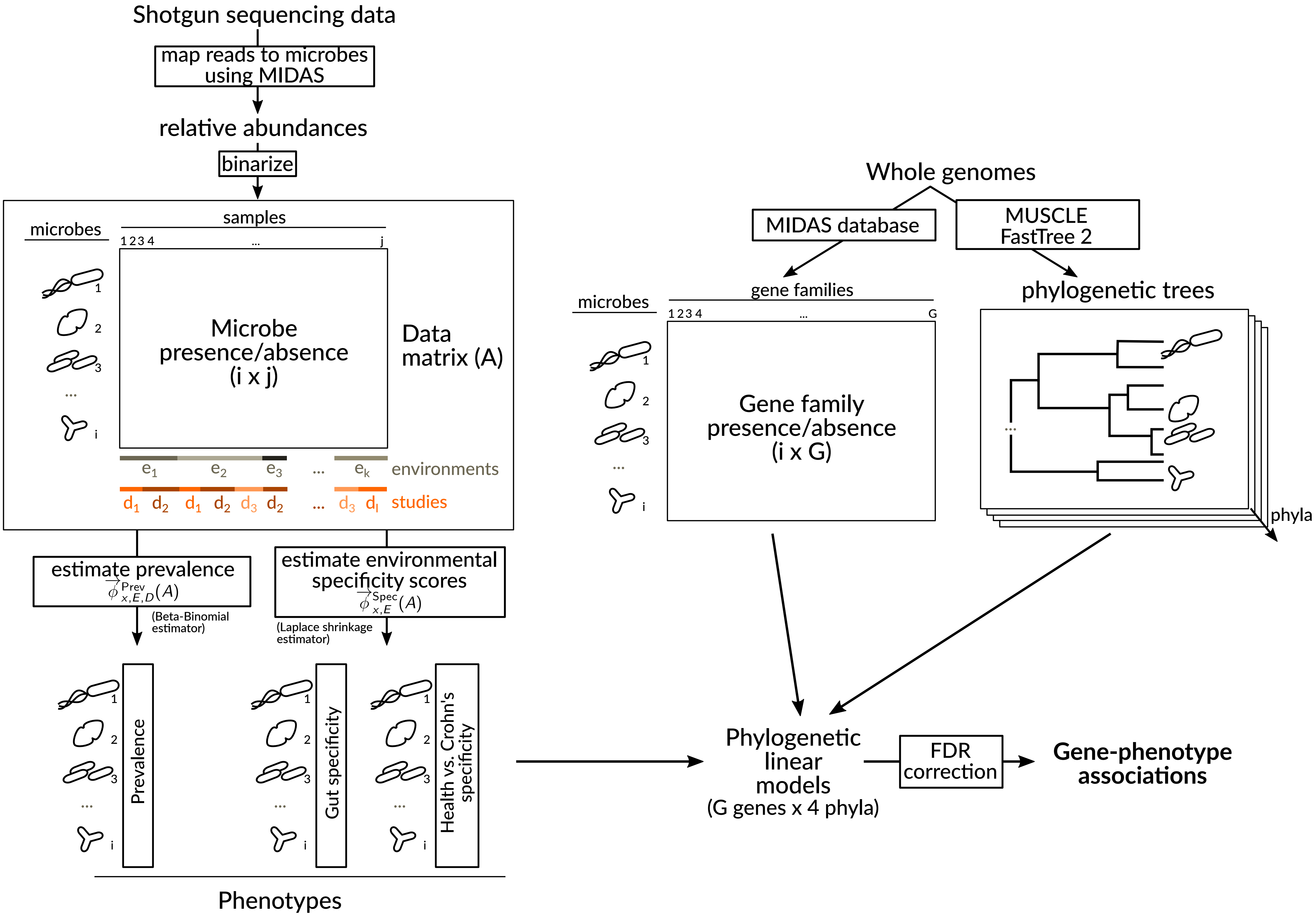

Shotgun sequencing data
map reads to microbes using MIDAS
relative abundances
binarize
microbes
samples
1 2 3 4 ... j
Microbe presence/absence (i x j)
Data matrix (A)
e1 e2 e3 ... ek environments
d1 d2 d1 d2 d3 d2 ... d3 dl studies
estimate prevalence
(Beta-Binomial estimator)
estimate environmental specificity scores
(Laplace shrinkage estimator)
Prevalence
Gut specificity
Health vs. Crohn's specificity
Phenotypes
Whole genomes
MIDAS database
MUSCLE FastTree 2
gene families
1 2 3 4 ... G
Gene family presence/absence (i x G)
phylogenetic trees
phyla
Phylogenetic linear models (G genes x 4 phyla)
FDR correction
Gene-phenotype associations
