# Supplementary material for: Phylogeny-corrected identification of microbial gene families relevant to human gut colonization
Source: PLoS Comput Biol. 2018 Aug 9;14(8):e1006242. doi: 10.1371/journal.pcbi.1006242 (PMC6084841; doi:10.1371/journal.pcbi.1006242)

A

	healthy	CD
present	1	1
absent	37	12

	healthy	CD
present	24	13
absent	14	0

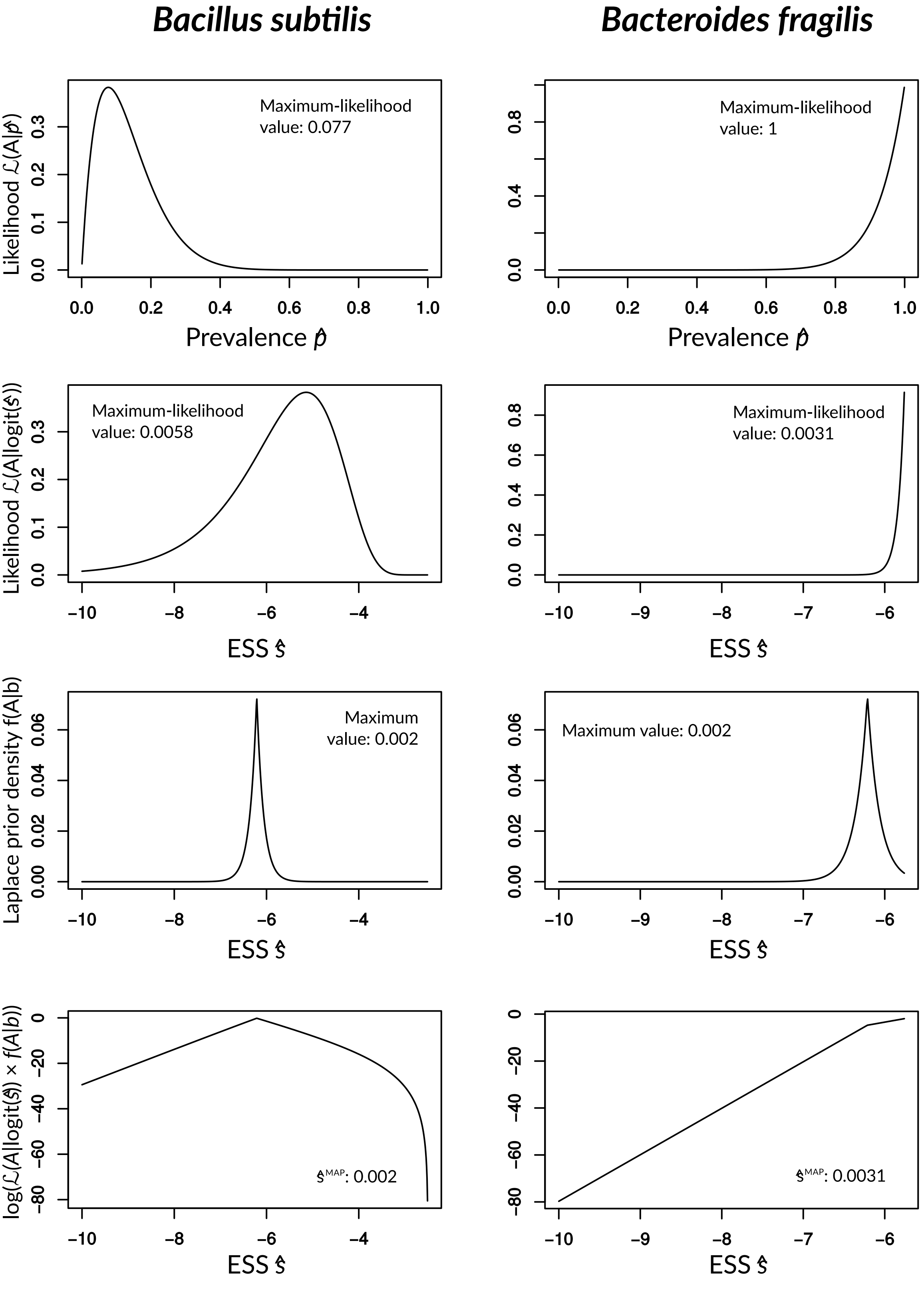

Supplement: S3 Fig — Two species are compared, one that was infrequently observed in both Crohn’s disease cases and controls (Bacillus subtilis, left) and one with a significant bias for Crohn’s disease cases (Bacteroides fragilis, right). A) Total counts across subjects for Bacillus subtilis and Bacteroides fragilis. B) Likelihood function for s^m,CD,E(A), or prevalence in Crohn’s disease. The maximum-likelihood value is given in the inset. C) Unregularized likelihood for logit(s^m,CD,E(A)), or the environmental specificity of the microbe. Note that the maximum-likelihood value (inset) was actually almost twice as large for Bacillus subtilis as for Bacteroides fragilis despite the relative paucity of data for B. subtilis (compare Y-axes, which show that the distribution for B. subtilis is flatter). D) Laplace prior around P(eCD) = 0.002 with width parameter b = 0.16 (optimized using simulation). E) Log-likelihood plot for the posterior P(eCD|m)=s^m,CD,EMAP(A), obtained by taking the product of the prior distribution and the unregularized distribution. The maximum a posteriori (MAP) estimates are the modes of these distributions (inset). (PDF) [file pcbi.1006242.s008.pdf]
