# Supplementary material for: Phylogeny-corrected identification of microbial gene families relevant to human gut colonization
Source: PLoS Comput Biol. 2018 Aug 9;14(8):e1006242. doi: 10.1371/journal.pcbi.1006242 (PMC6084841; doi:10.1371/journal.pcbi.1006242)
Supplement: S9 Fig — Lines are density plots of all pairwise correlations (either over all samples or over only non-zero values) for (A), the relative abundance of microbes within a simulation, and (B), the prevalence of simulated microbes across simulations with the same μm (mean) and zm (zero-inflation) values. A) Pairwise correlations for absolute abundances L(BiAbs) (black), column-normalized relative abundances L(BirelCN) (blue), and relative abundances obtained through Dirichlet-Multinomial sampling L(BirelDM) are shown, along with pairwise correlations over non-zero elements of relative abundances obtained through Dirichlet-Multinomial sampling LZ(BirelDM). B) Pairwise correlations for true prevalences L(ΠiAbs), for true prevalences above a sampling floor L(ΠiAbsThresh), and for prevalences calculated from relative abundances obtained through Dirichlet-Multinomial sampling L(ΠirelDM) are compared. (PDF) [file pcbi.1006242.s014.pdf]

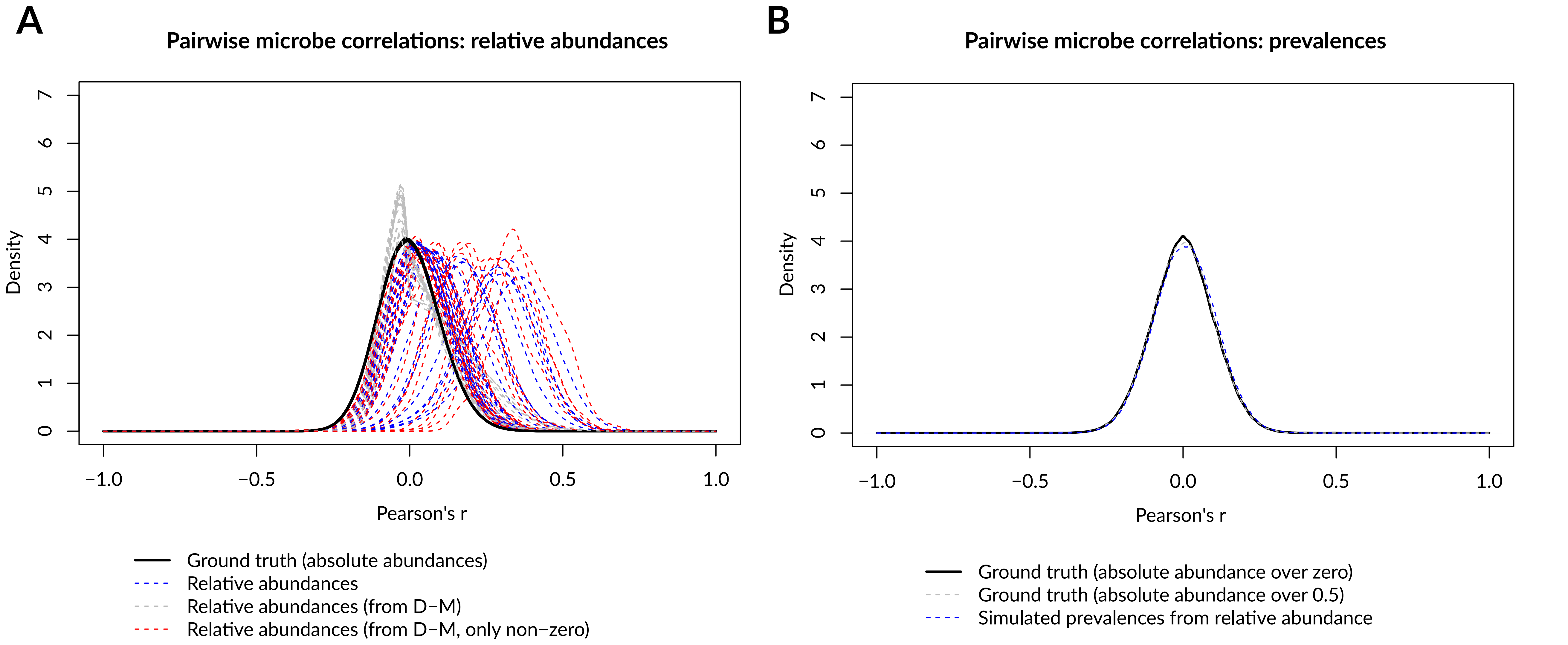

A
Pairwise microbe correlations: relative abundances
Density
Pearson's r
0
1
2
3
4
5
6
7
−1.0
−0.5
0.0
0.5
1.0
Ground truth (absolute abundances)
Relative abundances
Relative abundances (from D−M)
Relative abundances (from D−M, only non−zero)
B
Pairwise microbe correlations: prevalences
Density
Pearson's r
0
1
2
3
4
5
6
7
−1.0
−0.5
0.0
0.5
1.0
Ground truth (absolute abundance over zero)
Ground truth (absolute abundance over 0.5)
Simulated prevalences from relative abundance
